# WHAT DO FAMILY CAREGIVERS WANT? PAYMENT FOR PROVIDING CARE

**DOI:** 10.1093/geroni/igac059.3144

**Published:** 2022-12-20

**Authors:** Pamela Nadash

**Affiliations:** University of Massachusetts Boston, Boston, Massachusetts, United States

## Abstract

Although the primary goal of self-directed programs providing long term services and supports (LTSS) is to maximize choice and control for service recipients, such programs may also benefit family caregivers by compensating them for providing supportive services. This study draws on qualitative data from research supporting the RAISE Family Caregiver Advisory Council, finding that family caregivers themselves see the expansion of self-directed programs as a policy priority due to their need for financial security. The request for compensation was the strongest finding, with respondents highlighting the incompatibility of work with caregiving and their inability to rely on the existing paid workforce due to supply and quality issues; the consequences of this loss of earned income were reported as severe. Ultimately, respondents saw payment for providing care as an issue of fairness. This evidence supports the policy case for expanding access to self-directed programs that permit the employment of family caregivers.

